# An Early Warning System Based on Syndromic Surveillance to Detect Potential Health Emergencies among Migrants: Results of a Two-Year Experience in Italy

**DOI:** 10.3390/ijerph110808529

**Published:** 2014-08-20

**Authors:** Christian Napoli, Flavia Riccardo, Silvia Declich, Maria Grazia Dente, Maria Grazia Pompa, Caterina Rizzo, Maria Cristina Rota, Antonino Bella, The National Working Group

**Affiliations:** 1 National Centre for Epidemiology, Surveillance and Health Promotion, National Institute of Health (Istituto Superiore di Sanità, ISS), Viale Regina Elena, 299-00161 Rome, Italy; E-Mails: flavia.riccardo@iss.it (F.R); silvia.declich@iss.it (S.D.); mariagrazia.dente@iss.it (M.G.D.); caterina.rizzo@iss.it (C.R.); rota@iss.it (M.C.R.); antonino.bella@iss.it (A.B.); 2 Department of Prevention and Communication, Ministry of Health, Via Ribotta, 5-00144 Rome, Italy; E-Mail: m.pompa@sanita.it; 3 Coordinamento Gruppo Interregionale Sanità Pubblica e Screening, Via Dorsoduro, 3494/A-30123 Venezia, Italy; E-Mail: sanitapubblica.screening@regione.veneto.it

**Keywords:** migrant influx, early warning, syndromic surveillance, Italy

## Abstract

Profound geopolitical changes have impacted the southern and eastern Mediterranean since 2010 and defined a context of instability that is still affecting several countries today. Insecurity combined with the reduction of border controls has led to major population movements in the region and to migration surges from affected countries to southern Europe, especially to Italy. To respond to the humanitarian emergency triggered by this migration surge, Italy implemented a syndromic surveillance system in order to rapidly detect potential public health emergencies in immigrant reception centres. This system was discontinued after two years. This paper presents the results of this experience detailing its strengths and weaknesses in order to document the applicability and usefulness of syndromic surveillance in this specific context.

## 1. Introduction

Italy, historically a country of emigrants, has become the target of immigration only at the end of the 1970s. In those years, for the first time, the number of immigrants to Italy exceeded the number of emigrants. Initially a limited number of people migrated to Italy, mostly repatriates, housekeepers, and asylum seekers in transit to other European countries [1]. Since then, the number of immigrants has grown exponentially. According to the National Bureau of Statistics (ISTAT), as of January 2013, there were 4,387,721 foreign nationals legally residing in Italy. This number does not include “migrants lacking a regular status” [2]. In Italy, these include both migrants arriving without a regular visa and residence permit (clandestine migrants) and migrants who, having entered the country legally, have an expired residence permit that has not been renewed (irregular migrants) [3]. Over recent years, Italy has also experienced a growing influx of clandestine migrants arriving via sea. These migrants are normally received in equipped ports and, following an initial registration and health assessment, are transferred to immigration centres located across the country. There are several types of immigration centres in Italy including detention centres for migrants pending repatriation and non-detention centres which host asylum seekers until their case is cleared. All immigration centres are under the responsibility of the Italian Ministry of Interior which contracts private and public organizations to cater for all internal services including self-managed out-patient health services [4].

Since 2010, geopolitical instability has affected many countries of the southern and eastern Mediterranean. Prevailing insecurity combined with the reduction of border controls in affected countries of North Africa led, between 2011 and 2012, to major population movements in the region and migration surges in southern European countries such as Italy, Spain and Greece [5,6]. In the case of Italy, this exceptional migration flow occurred via sea converging on a small southern island called Lampedusa. In April 2011, following the declaration of a national humanitarian emergency, 20 Italian Regions and autonomous Provinces agreed to accept migrants transferred from Lampedusa, to host them in existing immigration centres and, if needed, to set up provisional immigration centres (by enrolling hotels and other private hosting structures).

In early April, a risk assessment of the potential public health impact on Italy of the migration surge was made by National Centre for Epidemiology, Surveillance and Health Promotion of the National Institute of Health (CNESPS-ISS) also thanks to bilateral interactions with concerned countries of the Mediterranean Region part of a network called EpiSouth [7]. This assessment clarified that, while people likely to arrive in Italy during the migration surge would be for the most part young adults in good health, living conditions within closed or semi-open communities (such as immigration centres) could expose them to communicable diseases. Rapid detection of potential public health emergencies among incoming migrants was recognized as a challenge, because they were hosted across Italy in a large number of formal and provisional immigration centres and, in the case of formal centres, were assisted by internal health services that were largely independent from the National Health System. For this reason, concerned health Authorities of the Italian Ministry of Health (MoH) and CNESPS-ISS, in coordination with the Italian Regions, decided to implement an *ad hoc* syndromic surveillance system in all active immigration centres receiving migrants from North Africa, aimed at an early detection of potential health emergencies in order to set up appropriate control measures. 

This methodology was chosen for its documented remarkable ability to adapt to rapidly shifting public health needs, and for its capability to rapidly provide surveillance information that can allow timely and appropriate public health responses [8]. Initially developed with a focus on bioterrorism, syndromic surveillance has been applied to a wide range of public health issues, using different data sources, to monitor specific syndromes (e.g., acute flaccid paralysis; influenza-like-illness *etc*.), as well as a wider range of aspecific conditions during mass gatherings, heat waves, floods, pandemics or natural disasters, such as the Icelandic ash cloud [8,9,10,11,12,13,14]. The cross-border usefulness of this instrument in enhancing the monitoring of some infectious diseases has also been demonstrated among residents of Mexican cities along the border with US [15].

To our knowledge, the use of this tool in the context of a migration surge at national level had never been documented. This paper presents the results of this activity and describes its strengths and weaknesses with the aim to document the applicability and usefulness of syndromic surveillance in this specific context.

## 2. Methods 

In April 2011, the MoH formally launched a syndromic surveillance protocol, studied for the specific case at hand (*ad hoc*), to be applied to all Italian immigration centres under the coordination of the CNESPS-ISS. This syndromic surveillance was never intended to substitute the existing epidemiological information flows and was designed to work in concert with the Italian statutory surveillance for infectious diseases. Based on a previous national experience of syndromic surveillance [16], and on adapted definitions from international official documents [17], 13 syndromes were defined as potentially indicative of events of public health concern (Table 1). 

**Table 1:** List of the 13 syndromes under surveillance.

No.	Name of the Syndrome
**1**	Respiratory tract disease
**2**	Suspected pulmonary tuberculosis
**3**	Bloody diarrhoea
**4**	Watery diarrhoea
**5**	Fever and rash
**6**	Meningitis/encephalitis or encephalopathy/delirium
**7**	Lymphadenitis with fever
**8**	Botulism-like illness
**9**	Sepsis or unexplained shock
**10**	Haemorrhagic illness
**11**	Acute jaundice
**12**	Parasite skin infection
**13**	Unexplained death

The syndromes’ definitions were reported in a previous publication [18]. The protocol, detailing implementation steps, the list of syndromes and their definition, was published and sent to all involved immigration centres. Due to the urgent need to rapidly set up the surveillance system, it was not possible to design and deliver specific training. 

### 2.1. Statistical Analysis

Data collected by the centres included the aggregate number of all new cases meeting each syndrome definition and the population hosted in the immigration centre by age group. The surveillance was paper-based: a standardized reporting form was used to collect information and was relayed every day from each immigration centre (or alternatively from local /regional health authorities) via email or fax to the CNESPS-ISS and to the MoH. The date of data collection and the reporting date were systematically recorded. Centres were asked to send forms with population data also on days when no new syndromes were being reported (zero reporting). Reported data were entered by CNESPS-ISS in a relational database developed in MS Access.

**Figure 1:**
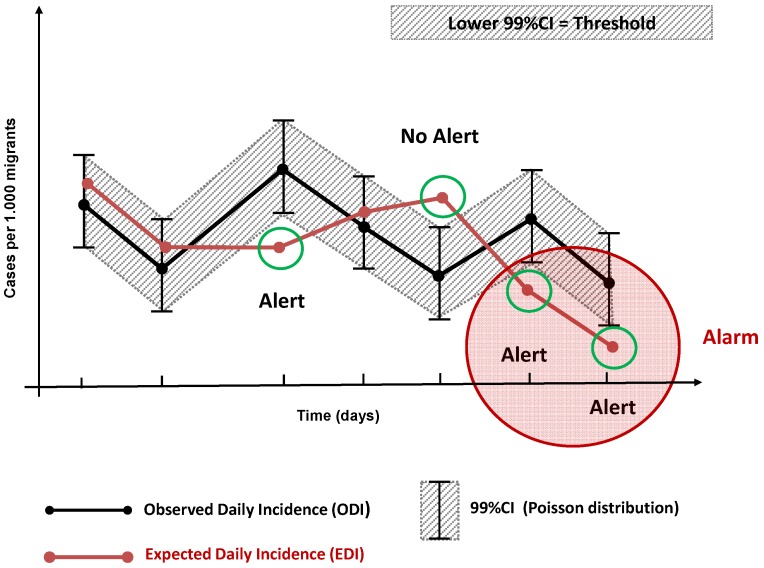
Statistical model for alerts and alarms identification.

The Observed Daily Incidence (ODI) was calculated by dividing the number of cases observed each day in the reporting immigration centres by the number of migrants present that same day. The moving average of the previous seven days incidence was used to define each syndromes’ Expected Daily Incidence (EDI). The EDI of each syndrome was measured against a threshold set at 99% confidence interval (99% CI) of the ODI using a Poisson distribution. A statistical alert was automatically triggered when the EDI fell outside this threshold (Figure 1). Statistical alerts were considered valid only when the EDI fell below the ODI (*i.e.*, when the observed incidence was higher than expected). A statistical alarm was issued whenever valid statistical alerts were triggered on the same syndrome for at least two consecutive days (Figure 1). Statistical analyses were carried out using STATA software version 11.2 (Stata Corporation, College Station, TX, USA).

Whenever a statistical alarm was issued, CNESPS-ISS team launched the same statistical analysis by reporting immigration centre to assess whether the incidence increase observed for a syndrome across all centres actually triggered statistical alerts and/or alarms at immigration centre level. If this was the case, ISS-CNESPS staff contacted the reporting health officer of the concerned centre/s by telephone and/or email to provide a detailed surveillance report and receive epidemiological feedback on the situation being faced. Based on the feedback received, the statistical alarm could be classified as unrelated to, or epidemiologically confirmed as an outbreak. When an outbreak was confirmed, follow up reporting on the ODI of the syndrome in the affected centre/s would be the responsibility of the ISS-CNESPS while local and regional health authorities with the support of the immigration centre health staff would have the responsibility of managing the outbreak response.

A national surveillance report with aggregated data was periodically published on the website of the CNESPS-ISS [19] and distributed to reporting health officers, MoH and all other concerned central and regional authorities. Timeliness of the surveillance system was monitored to assess if this tool was effectively meeting the objective of rapid outbreak detection. Therefore, monthly mean delays between data collection and reporting were assessed with the calculation of a 95% Confidence Interval (95% CI).

### 2.2. Period of Surveillance

After a preliminary period needed to recruit the immigration centres, to start the reporting and to build up the **EDIs**; the surveillance system officially started on the 1 May 2011. Although the humanitarian emergency was declared over on 31 December 2012, in order to permit the centres to enforce the statutory surveillance activities, the syndromic surveillance was extended to 30 June 2013. Therefore, the present study reports data acquired from 1 May 2011 to 30 June 2013.

## 3. Results

From the 1 May 2011 to the 30 June 2013, data were received from 139 immigration centres in 13 of 20 Italian Regions/Autonomous Provinces (AP). 

The mean number of immigrants under surveillance per day was 5362 (range: 1559‒8443). Overall, the population under surveillance was composed of adults aged between 25 and 44 years (40.6%), adolescents and young adults aged between 15 and 24 years (38.5%), children between 0 and 14 years (11.5%), and people over 45 (9.4%).

Overall, 7314 cases ascribable to the syndromes under surveillance were reported. Respiratory tract disease (49.0%), parasite skin infection (25.2%) and watery diarrhoea (22.6%) were the most commonly reported syndromes. No cases ascribable to botulism-like illness, sepsis or unexplained shock, haemorrhagic illness and unexplained death were reported (Table 2).

Two hundred and sixty statistical alerts were triggered by the surveillance system across all syndromes during its two years of activity, notably parasite skin infection (67), watery diarrhoea (59) and respiratory tract disease (45). Among those, 20 (7.7%) qualified as alarms for the following syndromes: respiratory tract disease (5), parasite skin infection (8), watery diarrhoea (5), suspected pulmonary tuberculosis (1) and bloody diarrhoea (1) (Table 2).

**Table 2:** Number of cases, alerts and alarms per syndrome, 1 May 2011–30 June 2013.

Syndrome	No. of Cases (%)	No. Alerts	No. Alarms
**1. Respiratory tract disease**	3586 (49.0)	45	5
**2. Suspected pulmonary tuberculosis**	76 (1.0)	33	1
**3. Bloody diarrhoea**	108 (1.5)	31	1
**4. Watery diarrhoea**	1652 (22.6)	59	5
**5. Fever and rash**	18 (0.2)	10	0
**6. Meningitis/encephalitis/encephalopathy/delirium**	2 (0.0)	1	0
**7. Lymphadenitis with fever**	27 (0.4)	11	0
**8. Botulism-like illness**	0	-	-
**9. Sepsis or unexplained shock**	0	-	-
**10. Haemorrhagic illness**	0	-	-
**11. Acute jaundice**	4 (0.1)	3	0
**12. Parasite skin infection**	1841 (25.2)	67	8
**13. Unexplained death**	0	-	-
Total	**7314**	**260**	**20**

**Figure 2:**
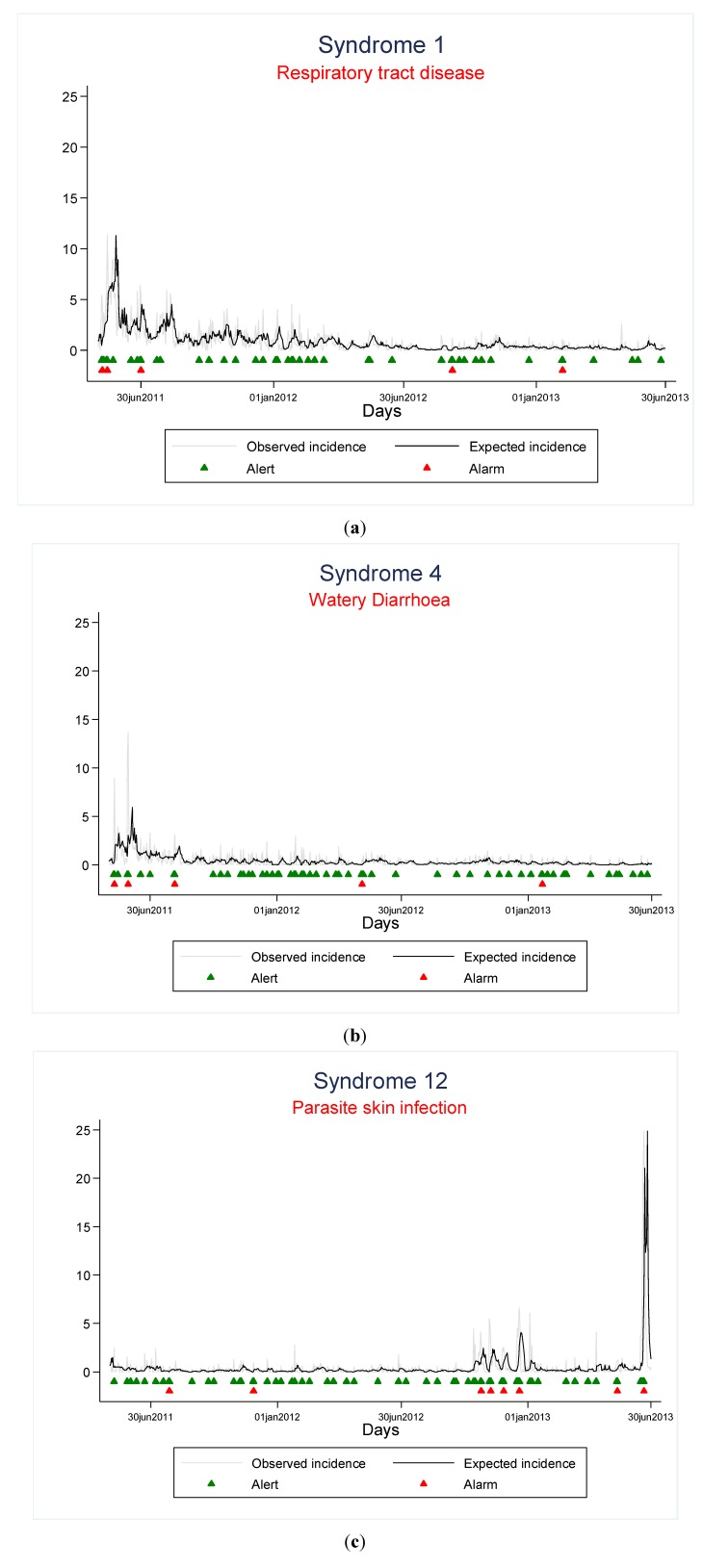
Alerts and alarms triggered by “Respiratory Tract Disease” (**a**), “Watery Diarrhoea” (**b**) and “Parasite Skin Infection” (**c**), 1 May 2011–30 June 2013.

Figure 2 shows the expected and observed incidence (per 1000 migrants) for the three most frequently reported syndromes.

All 20 statistical alarms were investigated. Most subsided within 24–72 h and were not triggered by a confirmed outbreak based on the feedback of the concerned immigration centre/s. An exception were three statistical alarms detected in November 2012, December 2012 and June 2013 for the syndrome “Parasite skin infection”, that were confirmed as scabies outbreaks taking place in one large immigration centre. Each one was controlled by the centre health authorities. The Positive Predictive Value (PPV) was 0.15 (95% CI: 0.11–0.19). 

**Figure 3:**
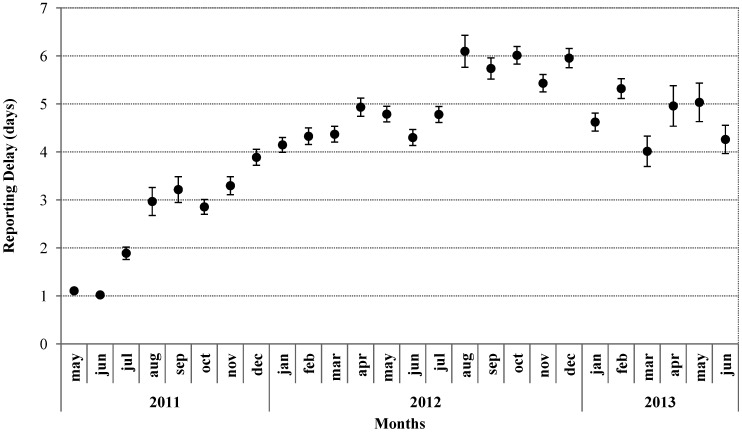
Timeliness of the syndromic surveillance system expressed as the mean delay per month in days and confidence interval (95% CI), 1 May 2011–30 June 2013.

Recruiting and fall out of centres occurred throughout the surveillance period, as new structures were made available and other closed. In particular, the number of provisional immigration centres, mostly hotels that hosted a very small number of migrants, notably changed during the surveillance period. Following an initial recruiting stage, data were received from an average of 20 immigration centres in the first 6 months of surveillance and over 30 immigration centres in the following 6 months. The mean signalling delay per month from the reporting centres to CNESPS-ISS was 4.4 days (Standard Error of the Mean ± 0.02) with average delays being below 3 days in the first four months of surveillance. Mean monthly delays increased progressively in the following months (Figure 3).

## 4. Discussion

With the arrival of thousands people suffering harsh travelling conditions in a very short time frame, the Italian migration surge of 2011 challenged Italian authorities. Moreover, an intense media reaction was triggered which needed accurate and reliable public health communication strategies. 

It also highlighted a need for cooperation among the different Italian authorities involved. The declaration of an emergency under Italian law implied the central role of the Italian Civil Protection (that is a Department under the Prime Minister’s Office) and of the Italian Ministry of Interior that coordinated through its offices on the ground (prefectures) the registration and transfer of migrants to the 20 Italian Regions that had agreed to host them. Each Region independently defined in which centres (existing or provisional) to accommodate migrants, allocated resources and in general managed the situation at local and intermediate level. 

This local and intermediate level independent management is also extended to the health sector because the Italian National Health System is highly decentralized too. The Ministry of Health, with the support of central technical bodies such as the ISS, has the responsibility to define with the Regional authorities the essential health services to be provided across the Country, the methods and the quality standards to be maintained and to issue ministerial decrees for aspects of national concern. But, Italian Regions enjoy autonomy in health care financing and delivery and are in charge of responding to public health concerns in their territorial units. 

In this context, an *ad hoc* surveillance system was set up using syndromic surveillance with the aim of supporting health care providers catering for migrants within immigration centres in rapidly detecting potential health treats. 

Although syndromic surveillance has been successfully implemented in a number of uncertain and high profile situations [8,9,10,11,12,13,14,15,16,20], its application has not been widely documented in the context of migration surges. 

There were several methodological difficulties that have been faced in implementing syndromic surveillance in this specific setting. Firstly, the target population was fluid: new arrivals were registered to Lampedusa nearly every day, and, with irregular frequency, migrants were transferred to other Italian Regions and between immigration centres. For this reason global, regional, local as well as centre based populations were extremely variable. In addition, while the first entry of each migrant was documented by the Italian Civil Protection to be national updated and rapidly available, once migrants were transferred within the country, responsibility shifted to the intermediate and local authority level and national official figures became very difficult to obtain.

Secondly, provisional centres were being fluidly opened and closed to reflect accommodation needs under the authority of Regions and some formal immigration centres suffered closures between 2011 and 2013 due to contingencies such as internal revolts. The exact number of nationally active centres at any time was variable and not generally available to health authorities. While new centres reporting were rapidly integrated as reporting units, it was not possible to determine *a priori* which centres were active but not reporting and, whether fluctuations in data arriving from centres were due to lack of adherence or to periodic closures. In addition, seven Regions known to be hosting migrants during the 2011 surge, never took part to the syndromic surveillance system. While follow up on discontinuously reporting centres was done on an *ad hoc* basis and data integrated when possible, assessing representativeness and completeness was not feasible. 

Thirdly, manual daily collection of data allowed for the rapid set up of the system and the creation of EDIs on the basis of weekly ODI data. However, as each centre had to compile and send syndromes and population data every day and data entry was manual and centralized within the CNESPS-ISS), this system was time and resource consuming at all levels. This reflected negatively on its sustainability, as shown by the timeliness of reporting that worsened following the initial months of surveillance.

Over the two years of continued surveillance, aside three isolated scabies clusters that were rapidly detected and controlled, the system documented the absence of major outbreaks. No additional outbreaks were documented in immigration centres during this period from other information sources. This confirmed that this migration flow was not associated with an increased risk of communicable disease transmission in Italy, as stated in the initial risk assessment and suggested by the surveillance preliminary findings [18]. Moreover syndromic surveillance provided updated information on the population hosted within reporting immigration centres and filled a potential communication gap by opening reporting channels between the medical staff working in immigration centres and public health officers. Improving communication between health care workers assisting individual patients and public health professionals aware of health issues at community level was considered one of the main added values of this experience. As all local health care workers were dedicated to the clinical assistance of individuals each in different centres with fluid populations and working on shifts, it was difficult for them to perceive empirically an increase in the number of cases of any disease. This was the case of the described scabies outbreaks that were detected as such through the systematic collection and analysis of data. Rapid detection allowed to put in place public health measures, in addition to individual treatments that were routinely prescribed. 

Syndromic surveillance applying Poisson distribution models was particularly suited to the migration surge scenario. This methodology works with fluctuating denominators, variable reporting units and with thresholds built in the absence of long term historical data trends that are common in this type of event. Once implemented in the context of the 2011 migration surge, this system was found to be effective and useful and, because of this, the surveillance system became a primary source of updated information. The choice of a more sensitive than specific syndromic approach led, as expected, to a low PPV. When automatically generated alerts and alarms were not triggered this could substantiate reassurance, while when this was not the case direct engagement with concerned reporting health officers enabled the rapid sharing of information on the evolving events. 

However, this system was not sustainable and presented limitations that need to be addressed before it can become a routine tool to tackle this type of event. Firstly, stronger coordination with different national institutional stakeholders is essential in order to have access to updated population and health centre data, as well as to encourage a wider adherence to the surveillance itself. This would improve the quality of the data collected and allow to calculate the system’s representativeness and completeness, providing a measure of the reliability of the information obtained. Secondly, as time and resources are precious during a migration surge, a surveillance system applied in this context should not be an excessive burden to health care providers and public health officials. The use of a web based secure platform enabling data entry at local level is needed to avoid duplication of activities, improve data quality and facilitate sustainability.

## 5. Conclusions

The critical analysis of this first experience in the use of syndromic surveillance during a migration surge has led to the elaboration of lessons learned that, confirming the usefulness of a syndromic surveillance system, are helping to shape an improved protocol to face the 2014 migration surge in Italy, that is expected to exceed the surge of 2011.
